# Predictors of psychiatric hospitalization among outpatients with bipolar disorder in the real-world clinical setting

**DOI:** 10.3389/fpsyt.2023.1078045

**Published:** 2023-03-16

**Authors:** Keita Tokumitsu, Norio Yasui-Furukori, Naoto Adachi, Yukihisa Kubota, Yoichiro Watanabe, Kazuhira Miki, Takaharu Azekawa, Koji Edagawa, Eiichi Katsumoto, Seiji Hongo, Eiichiro Goto, Hitoshi Ueda, Masaki Kato, Atsuo Nakagawa, Toshiaki Kikuchi, Takashi Tsuboi, Koichiro Watanabe, Kazutaka Shimoda, Reiji Yoshimura

**Affiliations:** ^1^Department of Psychiatry, Dokkyo Medical University School of Medicine, Tochigi, Japan; ^2^The Japanese Society of Clinical Neuropsychopharmacology, Tokyo, Japan; ^3^The Japanese Association of Neuro-Psychiatric Clinics, Tokyo, Japan; ^4^Department of Neuropsychiatry, Kansai Medical University, Osaka, Japan; ^5^Department of Neuropsychiatry, St. Marianna University School of Medicine, Kanagawa, Japan; ^6^Department of Neuropsychiatry, Keio University School of Medicine, Tokyo, Japan; ^7^Department of Neuropsychiatry, Kyorin University School of Medicine, Tokyo, Japan; ^8^Department of Psychiatry, University of Occupational and Environmental Health, Fukuoka, Japan

**Keywords:** bipolar disorder, predictor, real-world, outpatient, hospitalization

## Abstract

**Background:**

Bipolar disorder is a psychiatric disorder that causes recurrent manic and depressive episodes, leading to decreased levels of social functioning and suicide. Patients who require hospitalization due to exacerbation of bipolar disorder have been reported to subsequently have poor psychosocial functioning, and so there is a need to prevent hospitalization. On the other hand, there is a lack of evidence regarding predictors of hospitalization in real-world clinical practice.

**Methods:**

The multicenter treatment survey on bipolar disorder (MUSUBI) in Japanese psychiatric clinics was an observational study conducted to provide evidence regarding bipolar disorder in real-world clinical practice. Psychiatrists were asked, as part of a retrospective medical record survey, to fill out a questionnaire about patients with bipolar disorder who visited 176 member clinics of the Japanese Association of Neuro-Psychiatric Clinics. Our study extracted baseline patient characteristics from records dated between September and October 2016, including comorbidities, mental status, duration of treatment, Global Assessment of Functioning (GAF) score, and pharmacological treatment details. The incidence and predictors of hospitalization among patients with bipolar disorder over a 1-year period extending from that baseline to September–October 2017 were examined.

**Results:**

In total, 2,389 participants were included in our study, 3.06% of whom experienced psychiatric hospitalization over the course of 1 year from baseline. Binomial logistic regression analysis revealed that the presence of psychiatric hospitalization was correlated with bipolar I disorder, lower baseline GAF scores, unemployment, substance abuse and manic state.

**Conclusions:**

Our study revealed that 3.06% of outpatients with bipolar disorder were subjected to psychiatric hospitalization during a 1-year period that extended to September–October 2017. Our study suggested that bipolar I disorder, lower baseline GAF scores, unemployment, substance abuse and baseline mood state could be predictors of psychiatric hospitalization. These results may be useful for clinicians seeking to prevent psychiatric hospitalization for bipolar disorder.

## Introduction

Bipolar disorder is a psychiatric disorder with recurrent manic and depressive episodes (1–3). Bipolar disorder is classified into bipolar I disorder, which presents with manic episodes accompanied by severe psychosocial dysfunction, and bipolar II disorder, which presents with both hypomanic and depressive episodes, based on the DSM-5 criteria (3, 4). The annual prevalence of bipolar disorder is as high as ~2% (5), complications with other psychiatric and physical illnesses are reported, and there is an increased risk of suicide (5). Treatment is difficult. It has been noted that 30–60% of patients with bipolar disorder do not recover to their preonset level of functioning after achieving remission, and functional impairment has also been reported to be associated with high unemployment rates (6). A recent study has shown that the clinical course is a predictor of functional recovery, particularly that a high number of hospitalizations and being manic at baseline are predictors of subsequent worsening of functional impairment (7). In addition, concomitant use of psychotropic medications other than mood stabilizers has been found to be statistically significant as a predictor of both long-term hospitalization (8) and early readmission (9) in patients with bipolar disorder. On the other hand, a 1-year naturalistic cohort study at a single institution found no association between rehospitalization and any type of drug regimen, suggesting the equal effectiveness of individually optimized pharmacotherapy regimens for patients with bipolar disorder (10). There are also reports that bipolar I disorder is associated with a higher risk of psychiatric hospitalization than bipolar II disorder (11) and that comorbid substance use disorder is a predictor of psychiatric hospitalization (12). Furthermore, hospitalization and functional impairment in bipolar disorder are known to lead to increased social costs as well as patient distress (13). Therefore, preventing hospitalization, reducing the risk of recurrence of psychiatric symptoms, and improving patients' Global Assessment of Functioning (GAF) scores have been shown to be important from a health economic perspective for bipolar disorder (13). However, most studies on predictors of hospitalization for bipolar disorder have been single-center studies or reports of patients from Western countries. Therefore, there is insufficient real-world clinical evidence on the risk of hospitalization for patients with bipolar disorder in other regions including Asia.

In Japan, more than 90% of patients with mood disorders are outpatients, and approximately half of them is treated at clinics belonging to the Japanese Association of Neuro-Psychiatric Clinics (JAPC) (14). In this study, we conducted an observational study of outpatients with bipolar disorder in coordination with the Japanese Association of Neuro-Psychiatric Clinics and the Japanese Society of Clinical Neuropsychopharmacology (MUlticenter treatment SUrvey on BIpolar disorder in Japanese psychiatric clinics, or MUSUBI) (14–23). Since psychiatric clinics do not have inpatient beds, physicians affiliated with the clinics are highly interested in predictors of hospitalization in order to stabilize patients' mental status and prevent psychiatric hospitalization. We investigated the yearly rate of hospitalization and predictive factors for hospitalization among outpatients with bipolar disorder. This study is a *post-hoc* analysis using the same population as our previously published article (15).

## Subjects and methods

### Study design and subjects

The MUSUBI study was a retrospective study in which a questionnaire was administered at 176 outpatient clinics belonging to the JAPC with baseline patient characteristics data collected from September to October 2016 (14). We investigated the occurrence of psychiatric hospitalization over the course of 1 year extending from baseline to September–October 2017. The subjects used in this study are the same population as in our previously published article (15). Patients diagnosed with bipolar disorder based on the ICD-10 (2) and the DSM-5 (3) criteria and treated at these clinics were included in the study.

### Study procedures

Clinical psychiatrists were asked to complete a semistructured questionnaire on patients with bipolar disorder by performing a retrospective medical record survey. The questionnaire included patient characteristics (age, sex, height, weight, educational background, and work status), comorbidities, mental status, Global Assessment of Functioning (GAF) score, and details of pharmacological treatment as the baseline data. In addition, we assessed the occurrence of psychiatric hospitalization over the course of the year after baseline.

### Statistical analysis

All statistical analyses were performed with IBM SPSS Statistics (IBM Corporation, Version 28.0.0.0) and EZR (Saitama Medical Center, Jichi Medical University, Saitama, Japan) (24). The EZR provides a graphical user interface for R (The R Foundation for Statistical Computing, Vienna, Austria, version 4.0.3). More precisely, it is a modified version of R Commander (version 2.7–1) that incorporates statistical functions frequently used in biostatistics.

All statistical tests were two-sided with a significance level of 0.05. Demographic and clinical characteristics were analyzed using the chi-square test and the Student's *t*-test to identify differences between patients with and without psychiatric hospitalization over the course of the year after baseline. Bivariate analyses were performed to assess demographic and clinical features. In addition, the association between psychiatric hospitalization and the combination of prescriptions for mood stabilizers, antipsychotics, and antidepressants was analyzed with the chi-square test. All factors of demographic and clinical characteristics associated with the occurrence of psychiatric hospitalization among bipolar disorder patients were identified using binomial logistic regression with forced entry to avoid overlooking any potential associations. These factors included sex, body mass index (BMI), age at study entry, age at the onset of bipolar symptoms, current employment status, education level, mood stabilizer prescription (valproic acid, lithium carbonate, carbamazepine and lamotrigine), antipsychotic prescription, anxiolytic (benzodiazepine only) prescription, hypnotic prescription, intelligence quotient (IQ), GAF score, psychiatric comorbidities, personality disorder, developmental disorder, physical comorbidities, rapid cycling status, substance abuse (alcohol abuse), suicidal ideation, types of bipolar disorder and mood status. Intellectual disability was not included in developmental disabilities.

The following dummy variables of each factor were included in the binomial logistic regression analysis: male = 0, female = 1; employed = 1, unemployed = 0; educational background (special support education school or junior high school = 0, high school or vocational school = 1, junior college, technical college, university, master's degree or higher = 2); taking mood stabilizers = 1, not taking mood stabilizers = 0; taking antipsychotics = 1, not taking antipsychotics = 0; taking anxiolytics = 1, not taking anxiolytics = 0; taking hypnotics = 1, not taking hypnotics = 0; IQ (>85) = 0, IQ (<85) = 1; GAF (81–100) = 0, GAF (61–80) = 1, GAF (41–60) = 2, GAF (<41) = 3; psychiatric comorbidity = 1, no psychiatric comorbidity = 0; personality disorder = 1, no personality disorder = 0; developmental disorder = 1, no developmental disorder = 0; physical comorbidity = 1, no physical comorbidity = 0; rapid cycling = 1, no rapid cycling = 0; substance abuse (alcohol abuse) = 1, no substance abuse (no alcohol abuse) = 0; and suicidal ideation = 1, no suicidal ideation = 0. Four mood states (depressive, mixed, remission and manic/hypomanic) and types of bipolar disorders (bipolar I disorder, bipolar II disorder and bipolar disorder unclassifiable) were analyzed as nominal measures. A bipolar disorder not otherwise specified was listwise deleted; patients with serious physical conditions such as terminal cancer or intractable diseases were excluded. Cases with missing values in the questionnaire responses were also excluded. Other factors were not exclusionary for patients in this study.

## Ethics

This study was conducted in accordance with the Declaration of Helsinki and the Japanese Ethical Guidelines for Medical and Health Research Involving Human Subjects. Prior to the initiation of the study, the study protocol was reviewed and approved by the Institutional Review Board of the Ethics Committee of JAPC and Dokkyo Medical University School of Medicine (Approval No. 20160822, 2017-3). Since this was a retrospective medical record survey, it was exempted from the requirement for informed consent; however, we released information on this research so that patients were free to opt out. Administrative permissions and licenses were acquired by our team to access the data used. The ethics committee of the Japanese Association of Neuro-Psychiatric Clinics set restrictions on data sharing because of potentially identifying or sensitive patient information. Please contact the institutional review board of the ethics committee when requesting data. Contact information for our ethics committee is as follows: The Institutional Review Board of the Ethics Committee of the Japanese Association of Neuro-Psychiatric Clinics; Shibuya-ku, Yoyogi 1-38-2, Tokyo Metropolis, Japan, Postal Code 151–0053, Phone +81-3-3320-1423.

## Results

Representing baseline data, completed questionnaires on 3,137 outpatients with bipolar disorder were returned from the 176 originally solicited outpatient facilities. A total of 748 outpatients were excluded with listwise deletions due to missing values. Thus, data regarding a total of 2,389 patients were included in this study. The sociodemographic characteristics of the subjects are shown in Table 1.

**Table 1:** Demographic and clinical data for participants.

	**Psychiatric hospitalization for the next 1 year**		
**Factor**	**Hospitalization (–) (*N* = 2,316)**	**Hospitalization (+) (*N* = 73)**	***p*.value**
**Gender**			
Male: *n* (%)	1,067 (46.1)	28 (38.4)	0.233
Female: *n* (%)	1,249 (53.9)	45 (61.6)	
**Age at baseline [mean (SD)]**	50.42 (13.54)	51.22 (13.78)	0.622
**Age at onset [mean (SD)]**	34.78 (12.32)	32.81 (12.38)	0.177
**BMI at baseline [mean (SD)]**	23.43 (3.84)	23.03 (3.61)	0.386
**Bipolar disorder subcategories**			<0.001
Type I: *n* (%)	754 (32.6)	43 (58.9)	
Type II: *n* (%)	1,408 (60.8)	26 (35.6)	
Unclassifiable: *n* (%)	154 (6.6)	4 (5.5)	
**Mood status at baseline**			<0.001
Remission: *n* (%)	992 (42.8)	12 (16.4)	
Depressive state: *n* (%)	918 (39.6)	39 (53.4)	
Mixed feature: *n* (%)	224 (9.7)	9 (12.3)	
Manic/hypomanic state: *n* (%)	182 (7.9)	13 (17.8)	
**Work status at baseline**			<0.001
Unemployed: *n* (%)	744 (32.1)	45 (61.6)	
Employed: *n* (%)	1,572 (67.9)	28 (38.4)	
**Educational background**			0.978
Special support education school: *n* (%)	9 (0.4)	0 (0.0)	
Junior high school: *n* (%)	110 (4.7)	4 (5.5)	
High school, vocational school: *n* (%)	1,078 (46.5)	36 (49.3)	
Junior college, technical college: *n* (%)	212 (9.2)	7 (9.6)	
University: *n* (%)	828 (35.8)	24 (32.9)	
Master's degree or higher: *n* (%)	79 (3.4)	2 (2.7)	
**Mood stabilizer prescription**			
**Lithium carbonate**			0.122
No: *n* (%)	1,203 (51.9)	45 (61.6)	
Yes: *n* (%)	1,113 (48.1)	28 (38.4)	
**Other mood stabilizers**			0.032
No: *n* (%)	1,091 (47.1)	25 (34.2)	
Yes: *n* (%)	1,225 (52.9)	48 (65.8)	
**Antipsychotics prescription**			0.012
No: *n* (%)	1,120 (48.4)	24 (32.9)	
Yes: *n* (%)	1,196 (51.6)	49 (67.1)	
**Anxiolytics prescription**			0.217
No: *n* (%)	1,474 (63.6)	41 (56.2)	
Yes: *n* (%)	842 (36.4)	32 (43.8)	
**Hypnotics prescription**			0.052
No: *n* (%)	939 (40.5)	21 (28.8)	
Yes: *n* (%)	1,377 (59.5)	52 (71.2)	
**Antidepressants prescription**			0.47
No: *n* (%)	1,346 (58.1)	46 (63.0)	
Yes: *n* (%)	970 (41.9)	27 (37.0)	
**Intelligence quotient (IQ)**			0.502
>85: *n* (%)	2,212 (95.5)	69 (94.5)	
85–71: *n* (%)	81 (3.5)	4 (5.5)	
<71: *n* (%)	23 (1.0)	0 (0.0)	
**GAF score**			<0.001
81–100: *n* (%)	776 (33.5)	6 (8.2)	
61–80: *n* (%)	1,078 (46.5)	30 (41.1)	
41–60: *n* (%)	410 (17.7)	30 (41.1)	
1–40: *n* (%)	52 (2.2)	7 (9.6)	
**Psychiatric comorbidity**			0.223
No: *n* (%)	1,883 (81.3)	55 (75.3)	
Yes: *n* (%)	433 (18.7)	18 (24.7)	
**Personality disorder**			0.312
No: *n* (%)	2,184 (94.3)	67 (91.8)	
Yes: *n* (%)	132 (5.7)	6 (8.2)	
**Developmental disorder**			0.231
No: *n* (%)	2,169 (93.7)	66 (90.4)	
Yes: *n* (%)	147 (6.3)	7 (9.6)	
**Physical comorbidity**			0.122
No: *n* (%)	1,606 (69.3)	44 (60.3)	
Yes: *n* (%)	710 (30.7)	29 (39.7)	
**Rapid cycler**			0.087
No: *n* (%)	2,061 (89.0)	60 (82.2)	
Yes: *n* (%)	255 (11.0)	13 (17.8)	
**Substance abuse (alcohol abuse)**			0.001
No: *n* (%)	2,202 (95.1)	62 (84.9)	
Yes: *n* (%)	114 (4.9)	11 (15.1)	
**Suicidal ideation**			<0.001
No: *n* (%)	2,079 (89.8)	54 (74.0)	
Yes: *n* (%)	237 (10.2)	19 (26.0)	
**Psychotic symptoms**			0.042
No: *n* (%)	2,139 (92.4)	62 (84.9)	
Yes: *n* (%)	177 (7.6)	11 (15.1)	

The proportion of patients who experienced psychiatric hospitalization over the course of the year after baseline was 3.06% (73/2,389). The results of the bivariate analysis are shown in Table 1. Because 24 comparisons were made, a Bonferroni correction was applied, yielding a corrected significance criterion of *p* < 0.0021 in bivariate analyses. Based on this threshold, the occurrence of psychiatric hospitalization significantly differed among the groups stratified by GAF scores, suicidal ideation, employment status, types of bipolar disorders, mood status at baseline and substance abuse (alcohol abuse).

In addition, the combination of mood stabilizers, antipsychotics and antidepressants was not statistically significantly associated with psychiatric hospitalization in patients with bipolar disorder in this study (*p* = 0.171) (Table 2).

**Table 2:** Prescription pattern among antidepressant, mood stabilizer, and antipsychotic prescriptions.

**Factor**	**Hospitalization (–)**	**Hospitalization (+)**	***p*.value**
**Prescription pattern**	**(*****N*** = **2,316)**	**(*****N*** = **73)**	
No prescription	52	0	0.171
MS	573	13	
AD	86	3	
MS+AD	409	8	
AP	113	4	
MS+AP	608	29	
AP+AD	126	3	
MS+AP+AD	349	13	

MS, Mood stabilizer; AP, Antipsychotic; AD, Antidepressant.

A binomial logistic regression analysis revealed that there were significantly lower GAF scores in the group of patients with psychiatric hospitalization during the year after baseline [GAF 41–60, OR (95% CI) = 3.565 (1.203–10.565), *p* = 0.022; GAF 1–40, OR (95% CI) = 5.678 (1.485–21.703), *p* = 0.011]; the group also had a higher proportion of patients with bipolar I disorder rather than bipolar II disorder [OR = 2.449 (1.418–4.230), *p* = 0.001] as well as higher proportions of patients with unemployment [OR = 0.475 (0.275–0.821), *p* = 0.008], and substance abuse (alcohol abuse) [OR = 2.294 (1.090–4.827), *p* = 0.029]. The group of patients in a manic/hypomanic state [OR = 3.097 (1.271–7.547), *p* = 0.013] had significantly higher proportions of patients with psychiatric hospitalization than the group where patients were in a state of remission (Table 3). Multicollinearity between variables was not observed in our binomial logistic regression analysis with a forced entry model.

**Table 3:** Binomial logistic regression analysis of factors for psychiatric hospitalization among all patients.

**Variables in the equation**	**Coefficient**	**Standard error**	**Wald**	***p*.value**	**Odds ratio (95% confidence interval)**
**Gender (being female)**	0.452	0.296	2.323	0.127	1.571 (0.879–2.808)
**Age at baseline**	0.010	0.012	0.665	0.415	1.010 (0.986–1.035)
**Age of onset**	−0.013	0.013	0.911	0.340	0.987 (0.962–1.014)
**BMI at baseline**	−0.063	0.035	3.341	0.068	0.939 (0.877–1.005)
**Bipolar disorder subcategories (reference; Type II)**			10.501	0.005	
Type I	0.896	0.279	10.311	0.001	2.449 (1.418–4.230)
Unclassifiable	0.251	0.575	0.191	0.662	1.286 (0.417–3.965)
**Mood status at baseline (reference; remission)**			7.655	0.054	
Depressive state	0.480	0.398	1.458	0.227	1.616 (0.741–3.525)
Mixed feature	0.094	0.525	0.032	0.859	1.098 (0.392–3.075)
Manic/hypomanic state	1.131	0.454	6.191	0.013	3.097 (1.271–7.547)
**Work status at baseline**	−0.745	0.279	7.118	0.008	0.475 (0.275–0.821)
**Educational background (reference; special support education school, junior high school)**			0.540	0.763	
High school, vocational school	0.226	0.572	0.156	0.693	1.253 (0.409–3.843)
Junior college, technical college, or higher	0.389	0.606	0.411	0.521	1.475 (0.450–4.840)
**Mood stabilizers prescription**					
Lithium carbonate	−0.376	0.278	1.833	0.176	0.687 (0.398–1.183)
Other mood stabilizers	0.363	0.278	1.705	0.192	1.438 (0.834–2.479)
**Antipsychotics prescription**	0.233	0.272	0.734	0.392	1.263 (0.741–2.152)
**Anxiolytics prescription**	0.238	0.259	0.844	0.358	1.269 (0.763–2.110)
**Hypnotics prescription**	0.170	0.277	0.374	0.541	1.185 (0.688–2.040)
**Antidepressants prescription**	−0.273	0.278	0.968	0.325	0.761 (0.441–1.311)
**Intelligence Quotient (IQ)**	−0.378	0.587	0.414	0.520	0.685 (0.217–2.166)
**GAF score (reference; 81–100)**			8.517	0.036	
61–80	0.693	0.496	1.952	0.162	2.000 (0.756–5.291)
41–60	1.271	0.554	5.260	0.022	3.565 (1.203–10.565)
1–40	1.737	0.684	6.443	0.011	5.678 (1.485–21.703)
**Psychiatric comorbidity**	−0.258	0.485	0.283	0.595	0.772 (0.298–1.999)
**Personality disorder**	−0.003	0.610	0.000	0.997	0.997 (0.302–3.294)
**Developmental disorder**	0.525	0.598	0.770	0.380	1.690 (0.524–5.452)
**Physical comorbidity**	0.477	0.268	3.159	0.076	1.611 (0.952–2.725)
**Rapid cycler**	−0.068	0.344	0.039	0.844	0.935 (0.476–1.835)
**Substance abuse (alcohol abuse)**	0.830	0.380	4.780	0.029	2.294 (1.090–4.827)
**Suicidal ideation**	0.523	0.323	2.631	0.105	1.687 (0.897–3.175)
**Psychotic symptoms**	−0.110	0.378	0.084	0.772	0.896 (0.427–1.881)
**Constant**	−4.842	1.383	12.263	<0.001	

In addition, we stratified patients with bipolar I disorder and bipolar II disorder, and analyzed the predictors of hospitalization in each population using binomial logistic regression analysis.

The results showed that a manic/hypomanic state at baseline, unemployment and substance abuse (alcohol abuse) were predictors of psychiatric hospitalization in patients with bipolar I disorder (Table 4). In addition, baseline age and lower GAF score were predictors of hospitalization in patients with bipolar II disorder (Table 5). However, the clinical impact of baseline age in patients with bipolar II disorder as a predictor of hospitalization was considered minor because the effect size was very small (*p* = 0.017, OR = 1.055, 95% CI = 1.010–1.103).

**Table 4:** Binomial logistic regression analysis of factors for psychiatric hospitalization among patients with bipolar I disorder.

**Variables in the equation**	**Coefficient**	**Standard error**	**Wald**	***p*.value**	**Odds ratio (95% confidence interval)**
**Gender (being female)**	0.022	0.410	0.003	0.958	1.022 (0.457–2.284)
**Age at baseline**	−0.019	0.017	1.342	0.247	0.981 (0.949–1.013)
**Age of onset**	−0.014	0.019	0.538	0.463	0.986 (0.950–1.024)
**BMI at baseline**	−0.055	0.047	1.348	0.246	0.947 (0.864–1.038)
**Mood status at baseline (reference; remission)**			5.910	0.116	
Depressive state	0.216	0.520	0.173	0.678	1.241 (0.448–3.44)
Mixed feature	−0.007	0.711	0.000	0.992	0.993 (0.247–3.998)
Manic/hypomanic state	1.162	0.555	4.380	0.036	3.195 (1.077–9.48)
**Work status at baseline**	−0.989	0.400	6.123	0.013	0.372 (0.170–0.814)
**Educational background (reference; special support education school, junior high school)**			1.622	0.444	
High school, vocational school	1.083	1.112	0.949	0.330	2.953 (0.334–26.091)
Junior college, technical college, or higher	1.378	1.154	1.426	0.232	3.968 (0.413–38.099)
**Mood stabilizers prescription**					
Lithium carbonate	−0.391	0.368	1.123	0.289	0.677 (0.329–1.393)
Other mood stabilizers	0.167	0.377	0.197	0.657	1.182 (0.565–2.474)
**Antipsychotics prescription**	0.513	0.395	1.685	0.194	1.671 (0.770–3.627)
**Anxiolytics prescription**	−0.040	0.366	0.012	0.913	0.961 (0.469–1.968)
**Hypnotics prescription**	0.247	0.387	0.407	0.524	1.280 (0.600–2.731)
**Antidepressants prescription**	−0.034	0.389	0.007	0.931	0.967 (0.451–2.072)
**Intelligence Quotient (IQ)**	0.439	0.736	0.355	0.551	1.550 (0.366–6.562)
**GAF score (reference; 81–100)**			4.988	0.173	
61–80	0.130	0.600	0.047	0.828	1.139 (0.351–3.694)
41–60	0.379	0.703	0.291	0.590	1.461 (0.368–5.791)
1–40	1.386	0.788	3.093	0.079	3.997 (0.853–18.719)
**Psychiatric comorbidity**	−0.868	0.767	1.281	0.258	0.420 (0.093–1.887)
**Personality disorder**	0.180	0.923	0.038	0.846	1.197 (0.196–7.307)
**Developmental disorder**	0.047	1.031	0.002	0.964	1.048 (0.139–7.906)
**Physical comorbidity**	0.379	0.376	1.020	0.313	1.461 (0.700–3.050)
**Rapid cycler**	−0.486	0.486	0.998	0.318	0.615 (0.237–1.596)
**Substance abuse (alcohol abuses)**	1.005	0.472	4.531	0.033	2.732 (1.083–6.893)
**Suicidal ideation**	0.601	0.456	1.739	0.187	1.824 (0.747–4.459)
**Psychotic symptoms**	0.431	0.467	0.851	0.356	1.539 (0.616–3.847)
**Constant**	−2.037	2.061	0.977	0.323	

**Table 5:** Binomial logistic regression analysis of factors for psychiatric hospitalization among patients with bipolar II disorder.

**Variables in the equation**	**Coefficient**	**Standard error**	**Wald**	***p*.value**	**Odds ratio (95% confidence interval)**
**Gender (being female)**	0.984	0.569	2.992	0.084	2.675 (0.877–8.156)
**Age at baseline**	0.054	0.023	5.694	0.017	1.055 (1.010–1.103)
**Age of onset**	−0.020	0.022	0.845	0.358	0.980 (0.939–1.023)
**BMI at baseline**	−0.105	0.064	2.656	0.103	0.900 (0.794–1.022)
**Mood status at baseline (reference; remission)**			3.293	0.349	
Depressive state	0.389	0.757	0.263	0.608	1.475 (0.334–6.509)
Mixed feature	−0.217	0.977	0.049	0.825	0.805 (0.119–5.461)
Manic state	1.356	0.905	2.248	0.134	3.882 (0.659–22.870)
**Work status at baseline**	−0.402	0.456	0.779	0.377	0.669 (0.274–1.634)
**Educational background (reference; special support education school, junior high school)**			1.495	0.474	
High school, vocational school	−0.929	0.763	1.485	0.223	0.395 (0.089–1.760)
Junior college, technical college, or higher	−0.861	0.839	1.053	0.305	0.423 (0.082–2.189)
**Mood stabilizers prescription**					
Lithium carbonate	0.018	0.486	0.001	0.970	1.018 (0.393–2.639)
Other mood stabilizers	0.875	0.497	3.102	0.078	2.399 (0.906–6.355)
**Antipsychotics prescription**	−0.328	0.458	0.515	0.473	0.720 (0.294–1.766)
**Anxiolytics prescription**	0.403	0.450	0.803	0.370	1.496 (0.620–3.613)
**Hypnotics prescription**	0.293	0.490	0.359	0.549	1.341 (0.513–3.502)
**Antidepressants prescription**	−0.228	0.454	0.252	0.615	0.796 (0.327–1.937)
**Intelligence Quotient (IQ)**	−18.192	5,252.586	0.000	0.997	0.000 (0.000–0.000)
**GAF score (reference; 81–100)**			11.899	0.008	
61–80	1.679	1.123	2.236	0.135	5.362 (0.593–48.459)
41–60	3.212	1.195	7.217	0.007	24.818 (2.383–258.435)
1–40	−15.692	8,005.084	0.000	0.998	0.000 (0.000–0.000)
**Psychiatric comorbidity**	0.456	0.734	0.387	0.534	1.579 (0.375–6.652)
**Personality disorder**	0.382	0.931	0.169	0.681	1.466 (0.236–9.094)
**Developmental disorder**	0.709	0.919	0.595	0.440	2.032 (0.335–12.320)
**Physical comorbidity**	0.521	0.475	1.204	0.273	1.683 (0.664–4.266)
**Rapid cycler**	0.244	0.548	0.198	0.657	1.276 (0.436–3.739)
**Substance abuse (alcohol abuse)**	0.668	0.831	0.645	0.422	1.95 (0.382–9.945)
**Suicidal ideation**	0.587	0.534	1.211	0.271	1.799 (0.632–5.122)
**Psychotic symptoms**	−0.883	0.868	1.034	0.309	0.413 (0.075–2.268)
**Constant**	−7.680	2.419	10.078	0.002	

## Discussion

In a real-world Japanese clinical setting, we found that 3.06% of outpatients with bipolar disorder experienced psychiatric hospitalization over the course of the year after baseline. This proportion was lower than observed in previous studies examining predictors of rehospitalization of patients with bipolar disorder (9, 10). Our study participants were likely at relatively low risk of hospitalization because they included patients with no previous hospitalizations. In addition, predictors of psychiatric hospitalization within 1 year in outpatients with bipolar disorder included bipolar I disorder, having a low GAF score, being unemployed, having a co-occurring substance abuse (alcohol abuse), and having a baseline manic state. Of these findings, the most notable came from a comparison of odds ratios; GAF score had the greatest impact on predicting psychiatric hospitalization in patients with bipolar disorder, with lower GAF scores being associated with a statistically significant increase in the risk of hospitalization. The combination pattern of prescriptions for mood stabilizers, antipsychotics, and antidepressants was not found to be significantly associated with hospitalization of patients with bipolar disorder. The most common prescription pattern was mood stabilizers in combination with antipsychotics, followed by mood stabilizers alone. Therefore, it was considered that overall, pharmacotherapy was practiced in accordance with treatment guidelines for bipolar disorder (1). On the other hand, 2.2% of patients were not using any mood stabilizers, antipsychotics, or antidepressants, but these patients were not hospitalized. Because of the small proportion of patients that were followed up without medication in our study, further research is needed to determine whether patients with stable psychiatric symptoms can be followed up with psychotherapy alone.

We also found that lower baseline GAF scores were significantly associated with the occurrence of psychiatric hospitalization over the course of the year after baseline among outpatients with bipolar disorder. On the other hand, a previous study found that baseline cognitive dysfunction itself is a predictor of poor prognosis in patients with bipolar disorder (25). In addition, other researchers reported that hospitalized patients with bipolar disorder had difficulty improving their social functioning after discharge from the hospital (6). Since there is evidence that treatment for social cognitive dysfunction may improve the quality of life among patients with bipolar disorder by enhancing academic performance, work capacity, and social relationships (26), it is important to implement appropriate evidence-based pharmacotherapy (1) and structured psychotherapy (27) that may improve social functioning from the prehospitalization stage.

In addition, we have shown that bipolar I disorder has a statistically significantly higher risk of hospitalization occurrence than bipolar II disorder over a 1-year observation period. A previously reported large cross-sectional study in Sweden showed that bipolar I disorder was associated with more hospitalizations, lower psychosocial functioning, and lower educational attainment than bipolar II disorder (28). In addition, long-term observational studies have reported more frequent manic/hypomanic episodes in bipolar I disorder than in bipolar II disorder (29, 30). Based on these previous studies, the results of the present study suggest that bipolar I disorder is more likely to present with severe manic and psychosocial dysfunction than bipolar II disorder and is more likely to result in hospitalization.

We have also shown that the baseline manic state of patients with bipolar disorder is a predictor of psychiatric hospitalization over the course of the year after baseline. In bipolar disorder, manic symptoms are reported to be more likely to cause interpersonal problems and are also associated with increased suicide risk (26). In addition, repeated and prolonged manic episodes impair cognitive function (31), and cognitive dysfunction itself has been reported to be a predictor of poor prognosis in patients with bipolar disorder (25). Therefore, treatment strategies focused on manic symptoms and maintenance of remission are needed. For pharmacotherapy of manic symptoms in bipolar disorder, mood stabilizers except lamotrigine are recommended, although concomitant use of antipsychotics may be effective (1). On the other hand, patients with bipolar disorder presenting with manic or psychotic depression reportedly have poor treatment adherence due to decreased insight (32, 33). In particular, patients with comorbid bipolar disorder and substance use disorder have been found to have low rates of adherence in using mood stabilizers (34).

The proportion of substance abuse among bipolar I disorder patients was ~60%, while that of bipolar II disorder was also high at 48% (35). Furthermore, patients with bipolar disorder were found to be over six times more likely than the general population to suffer from substance abuse (35). Patients with comorbid bipolar disorder and substance use disorder are known to have poor treatment adherence, leading to relapses of bipolar disorder (36). Substance use disorders, including alcohol abuse, exacerbate symptoms of bipolar disorder and make it more difficult to treat (36). Therefore, it is critically important for these patients to receive integrated treatment for both diseases, which can improve treatment adherence and outcomes (35).

In the past, there was concern that antidepressant prescriptions for bipolar disorder increased the risk of manic episodes (37). In recent years, however, the combination of mood stabilizers and antidepressants and the prescription of antidepressants for bipolar II disorder have become more acceptable (1). We previously reported that the rate of prescribing antidepressants for patients with bipolar disorder is high in Japan, as in Western countries (14), and that antidepressant prescribing for patients with bipolar disorder was not a predictor of manic/hypomanic episodes during a 1-year observation period (15). This study showed that antidepressant prescriptions at baseline is not a statistically significant predictor of hospitalization among outpatients with bipolar disorder over the course of the year. This result confirmed previous studies on the tolerability of antidepressants in patients with bipolar disorder. Interestingly, adjunctive antidepressant therapy has also been shown to decrease the readmission rate of bipolar I depression and prolong the time to readmission during a 1-year observation period (38). A recent review also presented psychosocial therapies for the adjunctive treatment of bipolar disorder that led to increased medication adherence, improved manic symptoms, and improved GAF scores (39). However, prescribing antidepressants to patients with bipolar disorder is not entirely problematic, since the evaluation of antidepressants varies depending on the type of antidepressant and the subtype of bipolar disorder, and the risk of mania is still controversial. Therefore, it might be suggested that the concomitant use of appropriate psychosocial therapies, in addition to pharmacotherapy with attention to adverse events, can lead to improvements in affective symptoms and GAF scores as well as a reduction in the risk of psychiatric hospitalization.

The results of the current study also revealed that comorbid substance use disorders may be a predictor of hospitalization in patients with bipolar disorder. Prior research has reported that substance use disorders, such as alcohol abuse, increase the risk of developing bipolar disorder (40). Substance abuse is also known to be an important risk factor for poor treatment adherence in patients with bipolar disorder (32). Approximately 60% of patients with bipolar disorder are considered at least partially non-adherent to medications (41), and because discontinuation of mood stabilizers increases the risk of relapse, treatment strategies focused on improving adherence have attracted attention (41). Furthermore, not only are substance use disorders frequently comorbid with bipolar disorder, but it has already been reported that comorbid substance use disorders are a poor functional prognostic factor for bipolar disorder (42). In addition, substance use disorders are more common in bipolar I disorder than in bipolar II disorder (43), and patients with bipolar I disorder and substance use disorders have a more severe course (42). The Systematic Treatment Enhancement Program for Bipolar Disorder (STEP-BD) has also indicated that baseline substance use disorder is a predictor of manic episodes (44). Since the results of our study revealed that baseline manic state, substance use disorder, and bipolar I disorder are predictors of psychiatric hospitalization, we suggest that outpatient treatment focused on substance use disorder can improve psychiatric symptoms and social functioning and consequently prevent hospitalization.

One mechanism for the development of substance use disorders arises from the self-treatment hypothesis, in which patients begin to use psychoactive substances in a self-medicated manner to heal intolerable psychological distress (45). It has been proposed that when individuals begin to use psychoactive substances such as alcohol in response to psychosocial problems caused by manic and depressive emotional fluctuations in the hope of reducing anxiety and getting better sleep, a dependence is gradually formed, and substance use itself exacerbates psychiatric symptoms (45). Since social isolation is said to be behind substance use disorders (46), group psychotherapy, such as participation in self-help groups, as well as pharmacotherapy may be effective (46). It has been thought that pharmacotherapy and psychotherapy focusing on such substance use disorders could ultimately prevent exacerbations of bipolar disorder.

In addition, this study found that being unemployed was a predictor of hospitalization. This result suggests that social isolation may lead to the exacerbation of psychiatric symptoms. It has also been reported that the intensity of stigma and workplace exclusion for bipolar disorder is associated with unemployment (47). Therefore, we have thought that efforts to improve the work environment and provide support for the patients themselves would lead to an alleviation of their psychiatric symptoms. On the other hand, it is also possible that the patients lost their jobs because they were unable to continue working when social functioning deteriorated due to exacerbation of psychiatric symptoms. Although it is difficult to argue a causal relationship in this regard, further research is needed.

## Limitations

Our study has several limitations. The study was conducted only in clinics that belonged to the Japanese Association of Neuro-Psychiatric Clinics and consented to participate in the study, which may have population biased the composition of the participants. In addition, patient selection was not randomized and was a retrospective study, which may have led to selection bias in the participating population. In addition, our study found that manic state is an important predictor of hospitalization in patients with bipolar disorder, but we did not analyze the severity of manic state. Furthermore, previous hospitalizations and the number of hospitalizations were not tabulated, making it impossible to separate the risk of first-time hospitalization from the risk of rehospitalization. We also assumed alcohol to be the primary substance of interest for substance use disorders and did not distinguish it from other psych-dependent substances (e.g., cocaine, marijuana, methamphetamine).

Our study showed that five independent variables (low GAF score, bipolar I disorder, baseline manic/hypomanic state, unemployment, and substance use disorder) were predictors of psychiatric hospitalization in patients with bipolar disorder during a 1-year observation period. Previous studies have suggested an association between these independent variables, but our study design did not allow us to go as far as demonstrating a causal relationship.

## Conclusions

Our study revealed that 3.06% of outpatients with bipolar disorder experienced psychiatric hospitalization over the course of 1 year from baseline. Our study suggests that a low GAF score, bipolar I disorder, unemployment, comorbid substance use disorder (alcohol abuse), and baseline manic/hypomanic state could be predictors of psychiatric hospitalization.

## Data availability statement

The raw data supporting the conclusions of this article will be made available by the authors, without undue reservation.

## Ethics statement

The studies involving human participants were reviewed and approved by the Institutional Review Board of the Ethics Committee of the Japanese Association of Neuro-Psychiatric Clinics and Dokkyo Medical University School of Medicine. Written informed consent from the participants' legal guardian/next of kin was not required to participate in this study in accordance with the national legislation and the institutional requirements.

## Author contributions

KT, NY-F, and KW conceived the ideas. NA, YK, YW, KM, TA, KE, EK, SH, EG, and HU collected the data. KT, NY-F, MK, RY, AN, TK, TT, KS, and KW analyzed the data. KT wrote the original draft of the manuscript. NY-F and KS provided critical feedback. All authors revised the manuscript and approved the final version of the manuscript.
